# Comparing Objective and Subjective Measures of Parkinson's Disease Using the Parkinson's KinetiGraph

**DOI:** 10.3389/fneur.2020.570833

**Published:** 2020-11-05

**Authors:** Mei Knudson, Trine Hoermann Thomsen, Troels Wesenberg Kjaer

**Affiliations:** ^1^Department of Mathematics and Statistics, Carleton College, Northfield, MN, United States; ^2^DIS Copenhagen, Copenhagen, Denmark; ^3^Department of Clinical Neurophysiology and Neurology, Zealand University Hospital, Roskilde, Denmark; ^4^Department of Clinical Medicine, Faculty of Health, University of Copenhagen, Copenhagen, Denmark

**Keywords:** Parkinson's disease, PKG, subjective and objective data, motor symptoms, wearable device, activities of daily life (ADL), UPDRS, mathematical model

## Abstract

**Background:** Parkinson's disease (PD) is a neurodegenerative disease that can lead to impaired motor function and execution of activities of daily living (ADL). Since clinicians typically can only observe patients' symptoms during visits, prescribed medication schedules may not reflect the full range of symptoms experienced throughout the day. Therefore, objective tools are needed to provide comprehensive symptom data to optimize treatment. One such tool is the Parkinson's KinetiGraph^®^ (PKG), a wearable sensor that measures motor symptoms of Parkinson's disease.

**Objective:** To build a mathematical model to determine if PKG data measuring Parkinson's patients' motor symptoms can predict patients' ADL impairment.

**Methods:** Thirty-four patients with PD wore the PKG device for 6 days while performing their ADL. Patients' PKG scores for bradykinesia and dyskinesia, as well as their responses to a questionnaire asking if their ADL-level had been impacted by various motor symptoms, were used to build a multiple regression model predicting the patients' Movement Disorder Society-Unified Parkinson's Disease Rating Scale (MDS-UPDRS) part II scores.

**Results:** Calculation of bradykinesia score response to medication showed that using a dosage response time of 30 min yielded a greater bradykinesia response than when the response time was set to 40, 50, 60, 70, 80, or 90 min. The overall multiple regression model predicting MDS-UPDRS part II score was significant (*R*^2^ = 0.546, *p* < 0.001).

**Conclusion:** The PKG's ability to provide motor symptom data that correlates with clinical measures of ADL impairment suggests that it has strong potential as a tool for the assessment and management of Parkinson's disease motor symptoms.

## Introduction

Parkinson's disease (PD) is a neurodegenerative disease that affects more than 10 million people worldwide. The disease is characterized by motor symptoms such as tremor, rigidity, bradykinesia, motor complications such as dyskinesia, and non-motor symptoms such as cognitive difficulties (1).

In recent years, wearable sensors that detect motor symptoms have been used to monitor and manage the treatment of PD. These sensors can be worn on the body and use algorithms to determine the presence and severity of these symptoms. Currently, wearable sensors are capable of measuring several various Parkinsonian motor symptoms and complications, such as bradykinesia (slow movements and a decreased ability to move the body), dyskinesia (involuntary muscle movements), and tremors (2).

Parkinsonian patients may experience difficulty in recognizing and reporting their symptoms, and subjective recordings, such as surveys and diaries, can be prone to bias and inaccuracies (3). Thus, wearable sensors can provide objective symptom data that can help clinicians modify medications to more effectively manage symptoms of Parkinson's disease (4, 5). While studies involving these sensors often compare device results to clinical assessments, to our knowledge, no study so far has analyzed device results in conjunction with patients' subjective experiences regarding their motor symptoms (6–8).

This article aims to evaluate the potential of the Global Kinetics Corporation's Parkinson's KinetiGraph^®^ (PKG) wearable device in accurately monitoring motor symptoms of Parkinson's disease. Using a multiple regression model, PKG measurements of patients' motor symptoms were compared to patients' subjective experiences of their motor symptoms and to their score from the Movement Disorder Society—Unified Parkinson's Disease Rating Scale (MDS-UPDRS) part II, a validated scale for measuring motor aspects of experiences of daily living (9).

## Materials and Methods

### Patients

We examined 34 patients with mild to moderate Parkinson's disease (Hoehn and Yahr scale 2–3 in ON state) aged 50–75 years who had had symptoms of Parkinson's disease for 3–7 years and had no dementia. All patients met the United Kingdom Brain Bank diagnostic criteria for PD (10). The patients were recruited from the Movement Disorder Clinic of Zealand University Hospital in Roskilde, Denmark as well as from a recruitment notice in a magazine for members of the Danish Parkinson's Association. The patients were clinically assessed for the presence and nature of their motor symptoms before joining the study, and patients' motor symptoms and motor complications were clinically assessed after inclusion using the MDS-UPDRS part III and part IV (data not reported). Age, gender, disease duration, number of Parkinson's drugs taken, number of doses per day, and illness severity (Hoehn and Yahr scale) data were recorded.

A control group was not used as part of this study, but a previous study by Griffiths et al. (11) has shown that the PKG reports different bradykinesia and dyskinesia score distributions between control subjects and patients with Parkinson's disease.

### PKG Monitoring

The necessary permissions to use the PKG hardware were obtained from the copyright holders of the product (Global Kinetics Corporation).

Patients were asked to wear the accelerometer at home over a period of seven days, during which they performed their normal daily activities. Each patient wore the PKG device on his or her most affected side. The PKG device contains a rechargeable battery, a triaxial accelerometer, flash memory, and sensors that detect when the device is being worn (11). The PKG was programmed to vibrate to alert the patient that a dose of medication was due, and the patient confirmed the actual time of the dose by placing his or her thumb on the screen of the PKG.

The PKG accelerometer measured bradykinesia and dyskinesia levels during 2-min epochs from 5:00 a.m. to 11:00 a.m. This time period was chosen to specifically study the response to dopaminergic treatment in the morning, as many patients who experience bradykinesia have poor motor function before the first medication dosage has been administered, known as Early Morning OFF episodes (EMOs) (12).

The PKG's algorithms recognized bradykinesia as movements that have low acceleration and amplitude, with long intervals between movements. Dyskinesia was recognized as movements with normal acceleration and amplitude, but with shorter intervals that contained no movements (11). Using the algorithm, the PKG produced a bradykinesia score and dyskinesia score for each 2-min epoch, for a total of 180 data points per symptom per patient per day over the course of the 6-h period of monitoring (ibid.).

### MDS-UPDRS Part II

The MDS-UPDRS part II provides a clinical measure of ADL-impairment and has been shown to highly correlate with other disability rating scales (13). The rating system quantifies motor experiences of daily living using 13 self-assessed items (speech, saliva and drooling, chewing and swallowing, eating tasks, dressing, hygiene, handwriting, doing hobbies and other activities, turning in bed, tremor, getting out of a bed, car, or deep chair, walking and balance, freezing). A score was determined for each patient using the scale 0 = normal, 1 = slight, 2 = mild, 3 = moderate, and 4 = severe to assess each item, yielding an overall possible score range of 0 to 52. Results are summarized in Table 1.

**Table 1:** Demographic characteristics of the patients.

	**Mean**	**Standard deviation**
Age	66.44	6.05
Disease duration (years)	5.03	1.40
Hoehn & Yahr score	2.24	0.43
	**N**	**%**
Women	18	53
Men	16	47
Number of PD drugs = 1	2	5.9
Number of PD drugs = 2	20	58.8
Number of PD drugs = 3	12	35.5
Number of doses = 1, 2	10	29.4
Number of doses = 3, 4	24	70.6
	**Mean**	**Range**
UPDRS part II score	12.92	5–27

### Questionnaire

The questionnaire section was comprised of eight yes/no questions regarding four specific motor symptoms and complications: bradykinesia, dyskinesia, fluctuations, and early morning off-periods. Four of these eight questions asked the patient if they felt their ADLs were significantly impacted by the respective symptoms; these questions constituted the subjective component of the questionnaire. The remaining four questions asked if the patient had demonstrated the respective symptoms according to the PKG measurements; these questions were completed by the clinician, and constituted the objective component of the questionnaire. For each question, a response of “no” corresponded to a score of 0, and a response of “yes” corresponded to a score of 1. Thus, the overall score could take values from 0 to 8, where a low score would correspond to a low level of motor symptom impact on ADLs, and a high score would correspond to a high level of motor symptom impact on ADLs.

### Data Analysis

Data analysis was performed using Microsoft Excel's Data Analysis ToolPak and MATLAB R2019a.

Since most patients did not put on the PKG device until partway through the first day, only the data from the second through seventh day were used for data analysis. All PKG data collected within this timeframe were used; percent of time with immobility (PTI) was tested using an analysis of variance (ANOVA) test and was not shown to be significantly different among patients.

An essential measure of drug effect is the bradykinesia response to medication. To determine the time until the drug took effect, bradykinesia score changes were calculated using seven different “response times,” representing the time until the medication took effect. Clinical experiences and statements from the patients regarding medication effects suggested that most patients experienced the ON state 30- to 60-min after medication was administered; as a result, response times of 30, 40, 50, 60, 70, 80, and 90 minutes post-dosage were chosen to examine the time until maximum bradykinesia reduction.

To calculate the response to medication for the 30-min analysis, the average bradykinesia score from the “pre-medication” and the “post-medication” period were calculated for each patient. The “pre-medication” score was defined as the average bradykinesia score from the beginning of the measurement period (5:00 a.m.) until 30 min after the first dosage, and the “post-medication” score was defined as the average bradykinesia score from 30 min after the first dosage until the end of the measurement period (11:00 a.m.). If a patient took more than one dose of levodopa within the 5:00 a.m. to 11:00 a.m. time window, the analysis was only conducted over the period before the second dose was taken. Then, the difference between the pre-medication and post-medication averages was calculated to obtain a “BK change” score for each patient. The process was repeated for the 40-, 50-, 60-, 70-, 80-, and 90-min response time analyses.

For the questionnaire results, two-sample *t*-tests were conducted to determine if there was a significant difference in the overall average bradykinesia and dyskinesia scores of those who responded “yes” to a question vs. those who responded “no” to a question. These *t*-tests were conducted on the results of both the subjective questions, which asked patients about their experiences with Parkinson's symptoms, and the objective questions, which used PKG data to place patients in the “yes” group or the “no” group.

Multiple linear regression models were made to determine the impact of bradykinesia response, dyskinesia score, and questionnaire score (dependent variables) on the UPDRS part II score (independent variable), which measures the impact of motor symptoms on ADLs.

## Results

### Patient Data

Thirty-four patients fulfilled the required criteria and completed the data collection period. All patients received typical drug combinations of levodopa, dopamine agonists, MAO-B, and/or COMT inhibitors, with 32 out of 34 (94%) patients receiving levodopa. The clinical and sociodemographic characteristics of the sample are shown in Table 1.

### Dosage Response Time

Onset of bradykinesia improvement after levodopa intake is strongly correlated with plasma dopamine levels, so dosage response time was calculated by determining bradykinesia score change (14). Using seven time values representing time until medication response occurs (30, 40, 50, 60, 70, 80, 90 min), bradykinesia change was calculated in the manner described in the Data Analysis section. To account for the variability in medication response time that can occur on daily basis, the analysis was conducted on the averaged bradykinesia scores of all 34 patients over 6 days. Calculations showed that the largest change in bradykinesia score occurred when the medication response time was set as 30 min, with a decrease of 31.11 points in bradykinesia score (Table 2).

**Table 2:** Bradykinesia (BK) score change for seven dosage response times.

**Minutes**	**Pre-medication effect BK average**	**Post-medication effect BK average**	**BK change**
30	67.28	36.17	31.11
40	65.61	35.83	29.78
50	64.33	35.36	28.97
60	63.16	34.94	28.23
70	61.96	34.48	27.48
80	60.80	33.96	26.84
90	59.64	33.50	26.14

### Questionnaire Responses

Sixteen *t*-tests were conducted to determine if there was a significant difference in the overall average bradykinesia and dyskinesia scores of those who responded “yes” to a question vs. those who responded “no” to a question.

Four of the *t*-tests of average bradykinesia scores of responders were significant. Patients who experienced bradykinesia symptoms during more than 50% of waking time had significantly higher average bradykinesia scores than those who did not, and patients who reported that severe bradykinesia impacted their ADL also had significantly higher average bradykinesia scores than those who reported no impact. With regards to dyskinesia impact on ADL, patients who experienced dyskinesia symptoms during more than 50% of waking time had significantly lower bradykinesia scores than those who did not, and patients who reported that severe dyskinesia impacted their ADL also had significantly lower bradykinesia scores than those who reported no impact (Table 3).

**Table 3:** Mean overall bradykinesia scores of “yes” responders and “no” responders.

	**No**		**Yes**		
**Question**	**BKS**	**SD**	**BKS**	**SD**	***p*-value**
BK measured during more than 50% of waking time?	41.07	10.19	52.91	7.40	<0.001^***^
Does severe BK impact patient's ADL?	44.38	11.33	51.43	8.49	0.047^*^
DK measured during more than 50% of waking time?	53.36	6.19	40.26	10.63	<0.001^***^
Does severe DK impact patient's ADL?	49.16	9.58	36.40	10.81	0.019^*^
EMO (early morning off) measured?	48.99	6.17	48.10	11.14	0.832
Did the patient experience EMO?	50.31	9.39	46.32	10.69	0.257
Fluctuation time measured during waking time?	46.67	11.30	49.72	9.62	0.419
Do fluctuations impact patient's ADL?	48.06	10.16	49.50	10.35	0.685

* *p < 0.05*,

*** *p < 0.001*.

Three of the *t*-tests of average dyskinesia scores of responders were significant. Patients who experienced bradykinesia symptoms during more than 50% of waking time had significantly lower dyskinesia scores than those who did not, and patients who reported that severe bradykinesia impacted their ADL also had significantly lower dyskinesia scores than those who did not report an impact. Patients who experienced dyskinesia during more than 50% of waking time had significantly higher dyskinesia scores than those who did not (Table 4).

**Table 4:** Mean overall dyskinesia scores of “yes” responders and “no” responders.

	**No**		**Yes**		
**Question**	**DKS**	**SD**	**DKS**	**SD**	***p*-value**
BK measured during more than 50% of waking time?	12.60	8.11	3.94	3.05	<0.001^***^
Does severe BK impact patient's ADL?	10.46	8.27	4.85	4.62	0.016^*^
DK measured during more than 50% of waking time?	3.92	2.92	12.63	8.16	<0.001^***^
Does severe DK impact patient's ADL?	6.59	6.69	10.03	7.37	0.346
EMO (early morning off) measured?	3.23	2.03	8.04	7.31	0.077
Did the patient experience EMO?	5.52	5.50	8.47	7.68	0.206
Fluctuation time measured during waking time?	7.78	7.49	6.62	6.51	0.645
Do fluctuations impact patient's ADL?	8.59	7.87	5.20	4.84	0.147

* *p < 0.05*,

*** *p < 0.001*.

### Regression Modeling

Multiple regression analysis was performed with UPDRS part II score as the output variable, modeled by three input variables. The first input variable, called BK change, represents the change in the before-medication average bradykinesia score and the after-medication average bradykinesia score using a medication response time of 30 min. The second variable, called DK average, is the patient's overall average dyskinesia score over 6 days. The third variable, called subjective, is the sum of the responses to the eight questions in the questionnaire where, for each question, a response of “yes” equals 1, and a response of “no” equals 0. The results of the regression indicated that the model explained 54.6% of the variance and that the model was a significant predictor of UPDRS part II score [*F*_(3,30)_ = 12.033, *p* < 0.001].

The multiple regression equation predicting UPDRS score is:

$$\begin{array}{l} \text{UPDRS}=-\text{0}.112(\text{BK change})-\text{0}.297(\text{DK average})\\ \;+1.376(\text{subjective})+13.496 \end{array}$$

**Figure 1:**
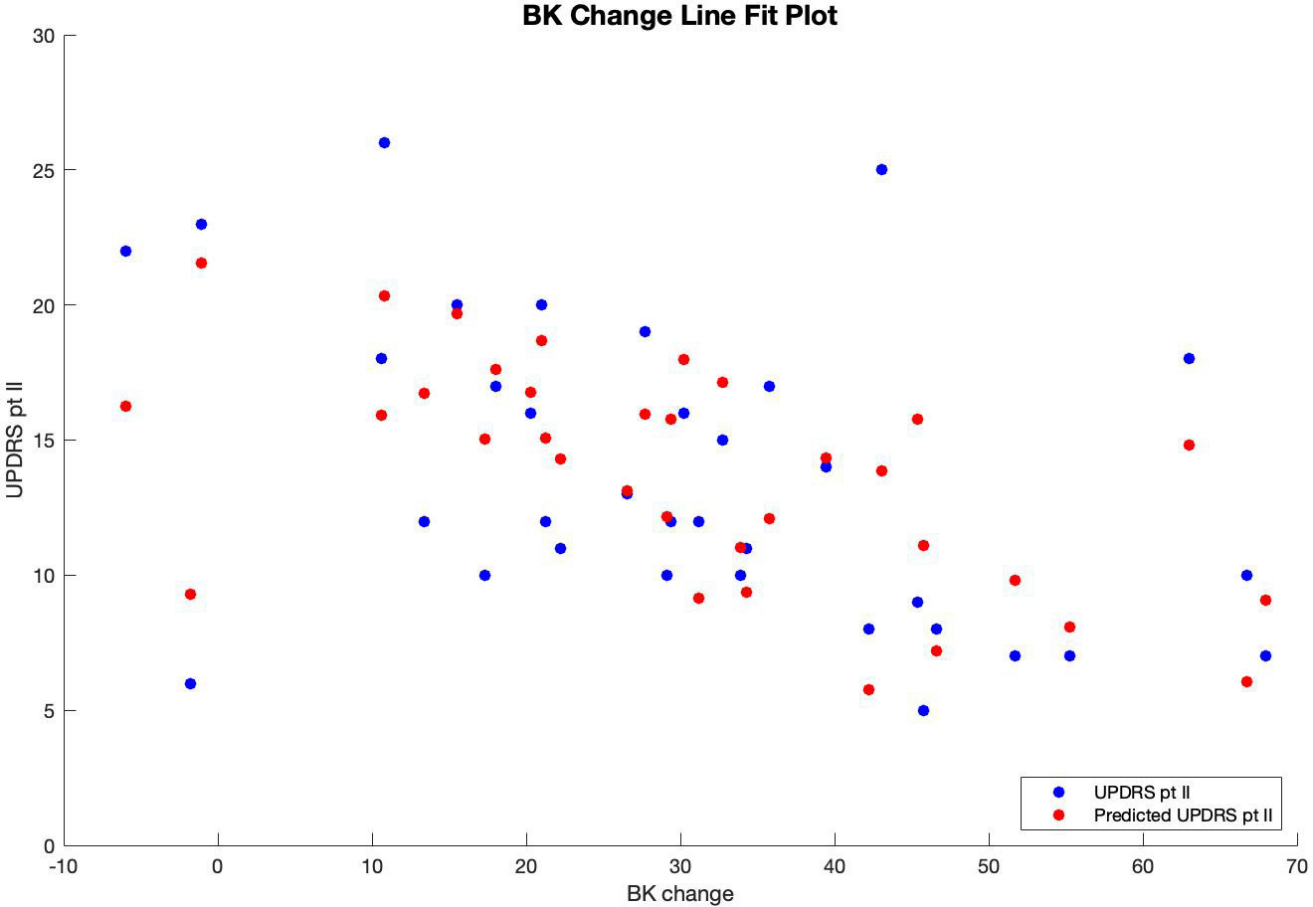
Line fit plot of bradykinesia response vs. UPDRS part II score.

**Figure 2:**
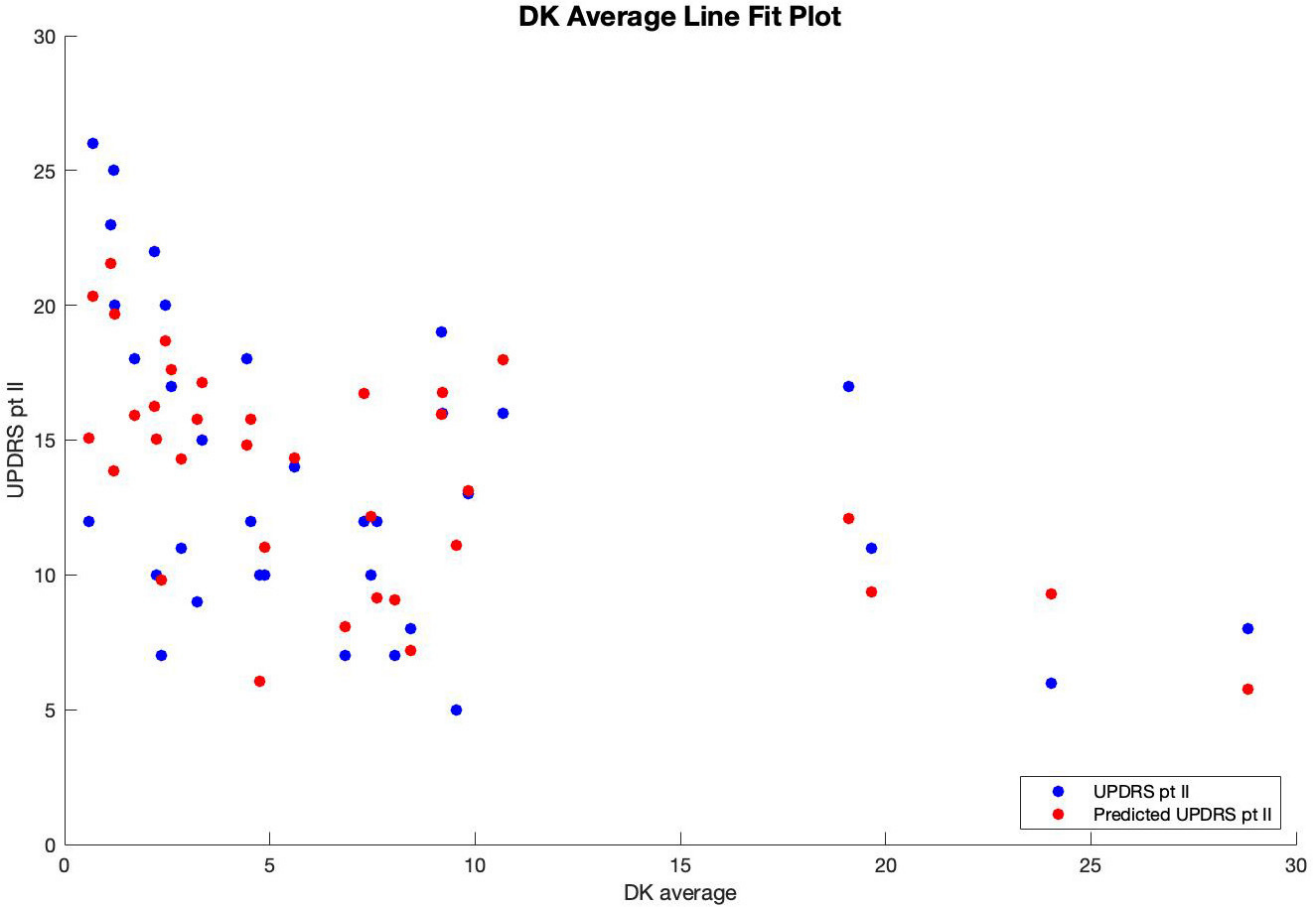
Line fit plot of average dyskinesia score vs. UPDRS part II score.

**Figure 3:**
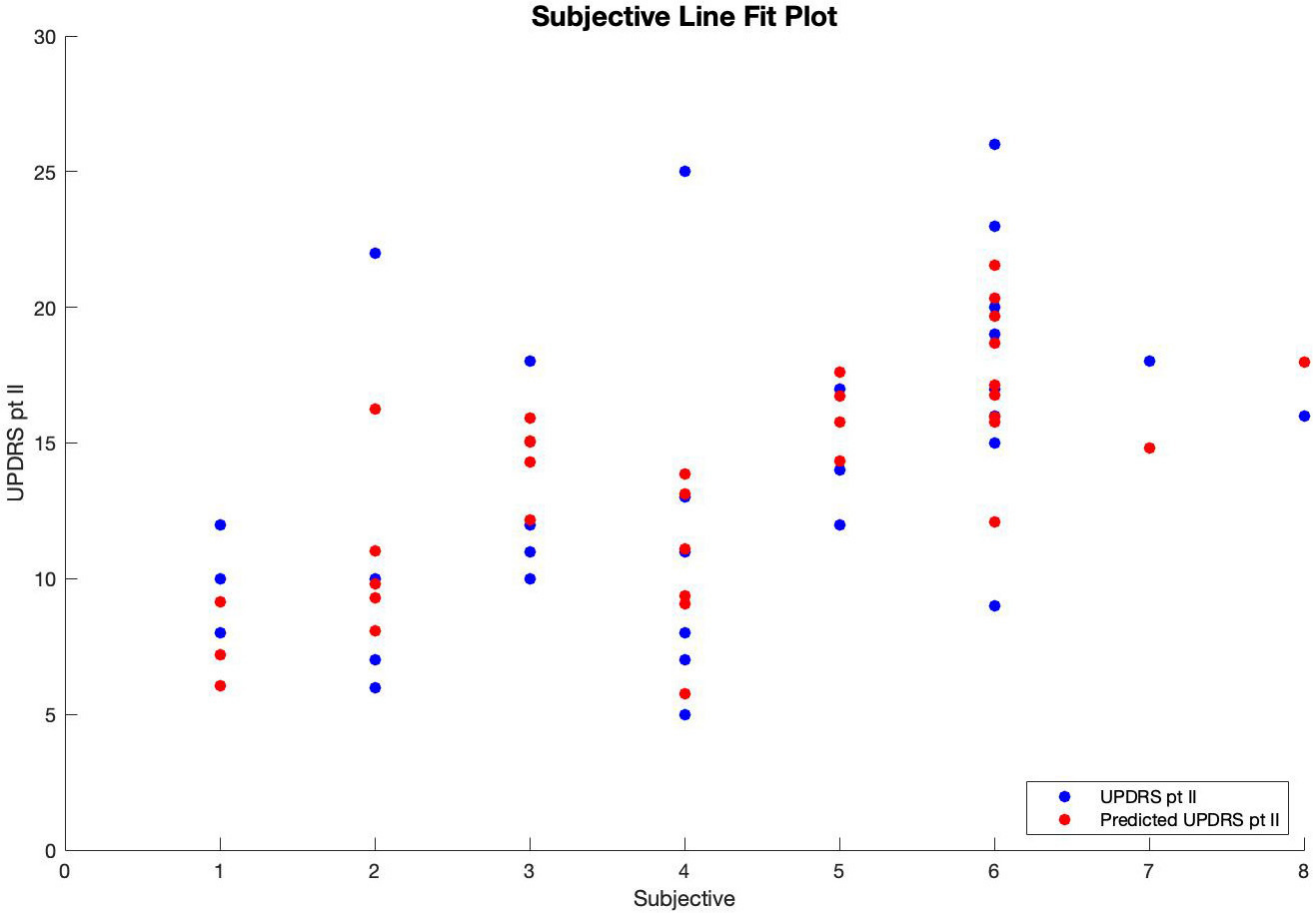
Line fit plot of subjective score vs. UPDRS part II score.

Therefore, patients' predicted UPDRS part II score was inversely correlated with their bradykinesia change score (Figure 1) and their dyskinesia score (Figure 2), and positively correlated with their subjective score (Figure 3). Specifically, UPDRS part II score decreased 0.112 points for each one-point increase in bradykinesia change, decreased by 0.297 points for each one-point increase in dyskinesia score, and increased by 1.376 points for each one-point increase in subjective score. All three coefficients had *p* < 0.05; the BK change coefficient had *p* = 0.006, the DK average coefficient had *p* = 0.007, and the subjective score coefficient had *p* = 0.0009. Therefore, each variable had a statistically significant effect on UPDRS part II score.

## Discussion

The main findings of this paper are as follows. First, the maximum reduction of bradykinesia symptoms occurred 30 min after intake of medication. Second, PKG data averages for 30-min bradykinesia change and overall dyskinesia, combined with patients' score on the eight-question subjective questionnaire, were used to create a significant multiple regression model predicting UPDRS part II score. The statistical significance of the model (*R*^2^ = 0.546) in predicting UPDRS part II score, a validated scale in predicting motor aspects of daily living (9), suggests that PKG measurements correlate with motor symptoms of Parkinson's disease.

### Dosage Response Time

Analysis of bradykinesia response to medication showed that there is a greater reduction of bradykinesia symptoms when a dosage response time of 30 min is used than when a dosage response time of 40–90 min is used. This result is a generalization based on the averaged responses of 34 patients taking different combinations of medications, and potential variations in patient responses as well as differences in patients' plasma drug concentration levels must also be considered. However, this metric could prove to be a useful baseline in determining the medication response time of individual patients. Several recent studies have shown that motor symptom data from wearable devices can help clinicians to better assess motor symptoms as well as to potentially alter medication schedule for better treatment and management of the disease (4, 15–17). Patients wearing a PKG or other similar wearable sensor could provide their motor symptom data to clinicians, who would be able to use sensor data in conjunction with this response time finding to determine the magnitude of the response to medication as well as the individual's expected response time.

### Two-Sample *t*-Tests

Two-sample *t*-tests yielded several significant results. The *t*-tests suggested that there is an inverse relationship between bradykinesia and dyskinesia; the average bradykinesia scores of those experiencing dyskinesia more than 50% of the time were significantly lower than those who did not, with *p* < 0.001, and the average dyskinesia scores of those experiencing bradykinesia more than 50% of the time were significantly lower than those who did not, with *p* < 0.001.

The inverse relationship between bradykinesia and dyskinesia can be attributed to the way in which levodopa dosages affect these symptoms. While levodopa treatment reduces bradykinesia symptoms by increasing dopamine levels, levodopa treatment can also increase dyskinesia symptoms in patients, especially when levodopa has been administered for a long period of time (18). Therefore, low average bradykinesia scores indicate that motor symptoms are well-treated with levodopa, but this is associated with the side effect of high average dyskinesia scores.

### Multiple Regression Analysis

The multiple regression analysis produced a model that had negative coefficients for the bradykinesia response and average dyskinesia variables, and a positive coefficient for the subjective score variable. This implies that a smaller (worse) bradykinesia response to medication, a lower average dyskinesia score, and a higher subjective score (high impact of motor symptoms on ADL) are each associated with a higher UPDRS part II score.

The bradykinesia response relationship to UPDRS part II score and the subjective score relationship to UPDRS part II score are not unexpected; patients who see less bradykinesia symptom improvement after taking medication would be expected to have higher levels of ADL impairment as measured by the UPDRS part II, and patients who report that they have a greater number of motor symptoms that impact their ADL would similarly be expected to have a higher UPDRS part II score. This result is in agreement with previous results that associate bradykinesia with lower quality of life (19). The inverse relationship between overall dyskinesia score and UPDRS part II score is also not unexpected, as previous studies have shown correlation between dyskinesia levels and quality of life (20, 21).

The overall regression model was significant, showing that data obtained from the PKG, combined with patients' subjective experiences of the impact of motor symptoms on their ADL, can provide an estimation of ADL impairment that is close to the actual UPDRS part II score. Significantly, this model incorporates both the patients' subjective experiences and their UPDRS part II score.

This study is, to the best of the authors' knowledge, the first PKG study to use a subjective questionnaire about motor symptoms as an evaluation tool. The significant result of the model suggests that the symptom data from the PKG can provide an accurate assessment of patients' overall level of impairment, and that the PKG has potential for use in evaluating and managing motor symptoms of Parkinson's disease. When used at home for extended periods of time, the data obtained from the PKG can give clinicians a more complete and realistic picture of a patient's experiences with motor symptoms, and aid in clinical decision-making (5, 22, 23).

### Limitations

One limitation in this study was the high amount of variability observed in the PKG data; for some patients, bradykinesia and dyskinesia averages would vary considerably between days. The presence of potentially outlying data points was attempted to be reduced by using more robust methods, such as averaging data over several days to produce a single score, but nonetheless may have negatively impacted the overall accuracy of the multiple regression model. This could be remedied by using a larger group of patients in order to minimize the influence of outliers. While the focus of this study was on response to dopaminergic treatment in the morning, results could also possibly be improved by recording PKG data throughout the day rather than just in the morning.

The high variability in PKG data also limited the dosage response time analysis. In particular, when calculating BK change for response times of fewer than 30 min, patients' average BK score for the pre-medication period would vary significantly on a day-to-day basis due to the small number of data points used to calculate the average pre-medication score. Due to the high degree of variance observed when calculating BK change for these short response times and clinical experiences with time until medication effect, only the response times of 30–90 min were reported. However, in order to verify our finding that a 30-min response time provided the greatest reduction in bradykinesia, it would be necessary to test additional response times.

Another potential limitation of this study is that two of the 34 patients did not receive levodopa, and only received agonist treatment. Exclusion of the two patients who only received agonist treatment may impact medication response time and model predictions, although previous statistical analysis on the same patient cohort showed that there was no significant difference between the number of PD drugs taken and the patients' MDS UPDRS part II score (*p* < 0.076) (24).

### Future Directions

In the future, similar studies could be done with a larger number of patients for a longer period of time so that data patterns would not be as strongly impacted by outliers. Experiments could also be done with patients who had more severe Parkinson's disease to see if the same results apply. In addition, PKG data could be used to predict different measures of disease severity and act as a “red flag” indicating the transition into the advanced phase of Parkinson's disease, thus enabling physicians to begin the appropriate treatment within a narrower timeframe. Hopefully, future studies will be able to supplement this study's findings about how wearable technologies can be used to both improve the quality of life of Parkinson's patients and clarify the relationship between management of ADL and response to medication.

## Data Availability Statement

The datasets presented in this article are not readily available because of GDPR regulations. Requests to access the datasets should be directed to trint@regionsjaelland.dk.

## Ethics Statement

The studies involving human participants were reviewed and approved by Danish Protection Agency (IBR: REG-110-2017) Regional Scientific Committee (IBR 58638). The patients/participants provided their written informed consent to participate in this study.

## Author Contributions

MK was responsible for the authorship of the manuscript, creation of figures and tables, and statistical analysis. THT was responsible for conducting the patient study, contributed literature references, and edited the article. TWK supervised the patient study, supervised the writing of the manuscript, and helped to edit the article. All authors contributed to the article and approved the submitted version.

## Conflict of Interest

The authors declare that the research was conducted in the absence of any commercial or financial relationships that could be construed as a potential conflict of interest.
